# Comparative genome analysis of enterohemorrhagic *Escherichia coli* ATCC 43894 and its pO157-cured strain 277

**DOI:** 10.71150/jm.2511015

**Published:** 2025-12-31

**Authors:** Se Kye Kim, Yong-Joon Cho, Carolyn J. Hovde, Sunwoo Hwang, Jonghyun Kim, Jang Won Yoon

**Affiliations:** 1 College of Veterinary Medicine & Institute of Veterinary Science, Kangwon National University, Chuncheon 24341, Republic of Korea; 2 Department of Molecular Bioscience, Multidimensional Genomics Research Center, College of Biomedical Science, Kangwon National University, Chuncheon 24341, Republic of Korea; 3 Department of Animal, Veterinary and Food Science, College of Agricultural and Life Sciences, University of Idaho, Moscow, Idaho 83844, USA; 4 Division of Zoonotic and Vector Borne Diseases Research, Center for Infectious Diseases, National Institute of Health, Cheongju 28159, Republic of Korea

**Keywords:** enterohemorrhagic *E. coli* O157:H7, whole genome sequencing, pangenome comparison

## Abstract

Enterohemorrhagic *Escherichia coli* (EHEC) O157:H7 ATCC 43894 (also known as EDL932) has been widely used as a reference strain for studying the pathophysiology of EHEC. To elucidate the role of a large virulence plasmid pO157 and its relationship with acid resistance, for example, both EHEC ATCC 43894 and its pO157-cured derivative strain 277 were well studied. However, it is unclear whether or not these two strains are isogenic and share the same genetic background. To address this question, we analyzed the whole genome sequences of ATCC 43894 and 277. As expected, three and two closed contigs were identified from ATCC 43894 and 277, respectively; two contigs shared in both strains were a chromosome and a small un-identified plasmid, and one contig found only in ATCC 43894 was pO157. Surprisingly, our pan-genome analyses of the two sequences revealed several genetic variations including frameshift, substitution, and deletion mutations. In particular, the deletion mutation of *hdeD* and *gadE* in ATCC 43894 was identified, and further PCR analysis also confirmed their deletion of a 2.5-kb fragment harboring *hdeD*, *gadE*, and *mdtE* in ATCC 43894. Taken together, our findings demonstrate that EHEC ATCC 43894 harbors genetic mutations affecting glutamate-dependent acid resistance system and imply that the pO157-cured EHEC 277 may not be isogenic to ATCC 43894. This is the first report that such genetic differences between both reference strains of EHEC should be considered in future studies on pathogenic *E. coli*.

## Introduction

Discovery and identification of reference strains in specific bacterial species provide a standard for functional studies and updating pharmacopoeias (An et al., 2022). Enterohemorrhagic *Escherichia coli* (EHEC) serotype O157:H7 is an etiological agent of bloody diarrhea and severe complication known as hemolytic uremic syndrome (Lee et al., 2023). The first two isolates of EHEC O157:H7 were collected from a 1983 hemorrhagic colitis outbreak in Michigan: EDL932 and EDL933. The whole genome sequence of EDL933 (ATCC 43895), a ground beef isolate, served as a reference for sequence comparison and a template for functional studies (Kim et al., 2022). EDL932, also known as ATCC 43894, was a patient isolate and its genome sequence was announced to serve as another reference in several studies (Lee et al., 2013; Wells et al., 1983). Genotypic variability was observed in ATCC 43894 compared to EDL933, such as a T-to-G mutation in 43894 *rpoS*, and these genetic variations among individual EHEC strains may confer different phenotypic traits (Uhlich et al., 2013, 2016).

Previously, ATCC 43894 displayed weak acid resistance whereas the strain 277, previously described as an isogenic pO157-cured isolate of ATCC 43894, was resistant to acid stress. Glutamate-dependent acid resistance (GDAR) associated genes were up-regulated in 277 but were significantly down-regulated in ATCC 43894 (Lim et al., 2007). Initially, these findings suggest pO157 involvement in GDAR regulation in EHEC O157:H7. Contrary to expectations, further analyses including re-introduction of pO157 to 277 revealed that pO157 does not harbor regulatory factors associated with GDAR (Wi et al., 2023), suggesting that there may be unidentified genetic elements associated with weak acid resistance in 43894. Here, we attempted to identify the genetic elements that confer difference in GDAR between ATCC 43894 and 277 using whole genome sequencing.

## Materials and Methods

### Bacterial strains

EHEC O157:H7 isolates in this study are ATCC 43894 and 277. EHEC ATCC 43894 is a laboratory stock, and 277 is a derivative strain cured of the virulence plasmid pO157 using acridine orange (Lim et al., 2007). These derivative strains are generous gifts from Carolyn Hovde. All bacterial strains were cultured in LB broth at 37°C for 19 h with shaking (230 rpm), or on LB agar plates at 37°C for 19 h without shaking.

### Whole genome sequencing and analysis

Genomic DNA samples from EHEC strains were extracted with HiYield Genomic DNA Mini Kit (RBC Bioscience, Taiwan) using the instructions provided by the manufacturer. DNA concentrations were measured, and two DNA samples (43894 and 277) were sent to a commercially available company (Macrogen, Korea) for whole genome sequencing.

Library preparation for PacBio or Illumina was done using SMRTbell^®^ Prep Kit (Pacific Biosciences, Inc., USA) or TruSeq PCR-free DNA High Throughput Library Prep Kit (Illumina, USA) using the manufacturers’ instructions. The whole genome sequencing was performed on both Pacific Biosciences SequelII and Illumina NovaSeq. The genome assembly was performed using a hybrid approach with Illumina and PacBio data. Briefly, both types of raw data were assembled using Unicycler v0.5.1 (Wick et al., 2017), and the resulting contigs underwent error correction with Pilon v1.24 utilizing Illumina data (Walker et al., 2014). The generated contigs were completed through manual curation, and genome annotation was conducted using Prokka v1.14.6 (Seemann, 2014). Comparative analysis between the two genomes was carried out by BLASTn. The whole genome sequencing raw data and annotations were deposited in the National Center for Biotechnology Information GenBank under BioProject PRJNA1251837 (BioSamples SAMN48025411 and SAMN48025412).

### Polymerase chain reaction amplification (PCR)

Oligonucleotides used in the amplification of the *hdeD*-*gadE*-*mdtE* fragment are the following: a forward (5′-CTG GCA GAA GAA GCA GAC CA-3′) primer and a reverse (5′-AAT CGG GTC CAG ACG TTG TA-3′) primer. PCR was performed with the following conditions: initial denaturation at 94°C for 5 min; 30 cycles of denaturation (94°C, 30 s), annealing (58°C, 30 s) and elongation (72°C, 1 min 30 s) steps; and final elongation at 72°C for 5 min. The gDNA extracted from 43894 and 277 were used as templates. Amplification was confirmed by electrophoresis in a 1% agarose gel and staining with GreenStar Nucleic Acid Staining Solution I (Bioneer, Korea).

### TA cloning and sequencing

TA cloning of the amplified PCR products was performed using T&A Cloning Kit (RBC Bioscience) in accordance with the manufacturer’s instructions. Sanger sequencing of the ligated products was performed commercially (Macrogen).

## Results

### Whole genome sequencing of ATCC 43894 and 277

**Fig. 1.**
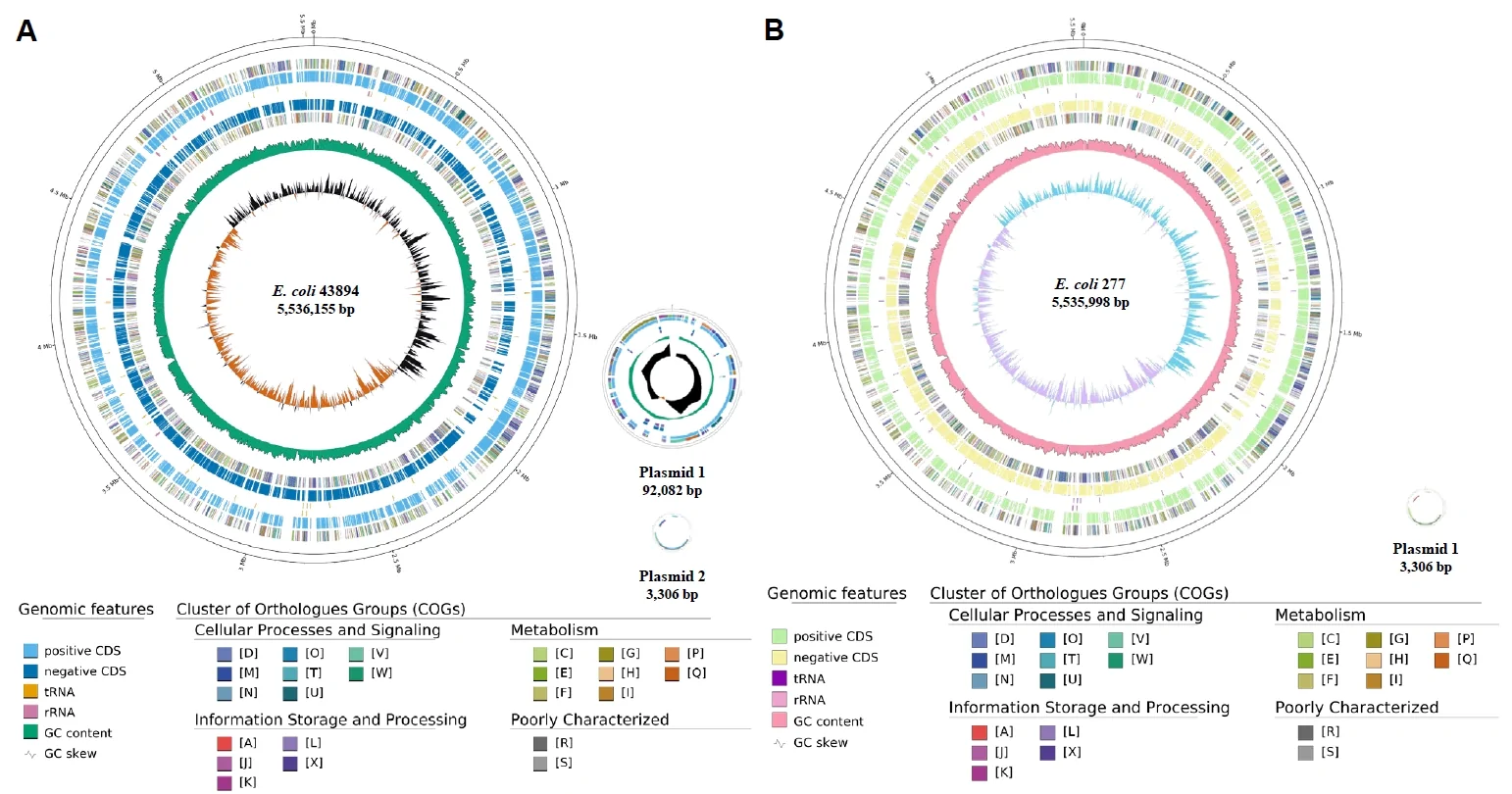
Complete genomes of EHEC O157:H7 strains 43894 and 277. Circular maps of three contigs from 43894 (A) and 2 contigs from 277 (B). Each circular map was drawn by applying the contig annotation information. Marked genomic features are shown from outside to inside; Clusters of Orthologues Groups (COG), positive coding sequence (CDS), negative CDS, tRNA, rRNA, G + C ratio and G + C skew. COGs are shown in colors in accordance with the COG categories: D, Cell cycle control, cell division, chromosome partitioning; M, Cell wall/membrane/envelope biogenesis; N, Cell motility; O, Posttranslational modification, protein turnover, chaperones; T, Posttranslational modification, protein turnover, chaperones; U, Intracellular trafficking, secretion, and vesicular transport; V, Defense mechanisms; W, Extracellular structures; A, RNA processing and modification; J, Translation, ribosomal structure and biogenesis; K, Transcription; L, Replication, recombination and repair; X, nan; C, Energy production and conversion; E, Amino acid transport and metabolism; F, Nucleotide transport and metabolism; G, Carbohydrate transport and metabolism; H, Coenzyme transport and metabolism; I, Lipid transport and metabolism; P, Inorganic ion transport and metabolism; Q, Secondary metabolites biosynthesis, transport and catabolism; R, General function prediction only; S, Function unknown.

We first performed the whole genome sequencing for ATCC 43894 and 277. As shown in Fig. 1, a total of three contigs with 5,631,543 bases were obtained from de novo assembly of ATCC 43894 genome: a chromosome (5,536,155 bp) and two plasmids (92,082 and 3,306 bp). The number of coding sequences (CDSs), ribosomal RNAs (rRNAs), and transfer RNAs (tRNAs) were 5419, 22, and 104, respectively. The G + C ratio of 43894 gDNA was 50.42% (chromosome, 50.47%; plasmid 1, 47.64%; plasmid 2, 43.41%). As for the strain 277 genome, two contigs with 5,539,304 bases were obtained from de novo assembly: a chromosome (5,535,998 bp) and a plasmid (3,306 bp). The number of CDSs, rRNAs, and tRNAs were 5328, 22 and 104, respectively; the fewer CDSs in 277 reflect the number of missing CDSs from pO157. The G + C ratio of 277 gDNA was 50.46% (chromosome, 50.46%; plasmid 1, 43.41%), similar to that of 43894 gDNA. The minute difference may be due to the presence of the virulence plasmid pO157 in 43894, as its presence lowered the overall G + C ratio. Collectively, the whole genome sequencing revealed that EHEC O157:H7 strains ATCC 43894 and 277 are sequentially similar to one another, and that 277 is indeed cured of the virulence plasmid pO157.

### Pan-genomic comparison between ATCC 43894 and 277

**Fig. 2.**
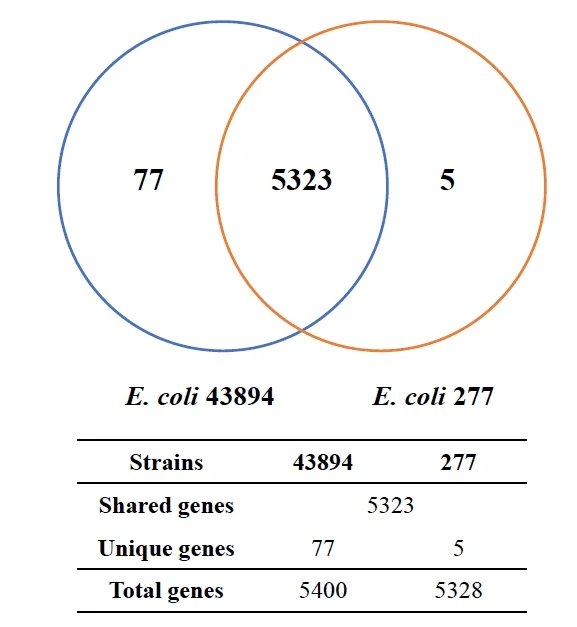
Pangenome comparison of EHEC O157:H7 strains 43894 and 277. A Venn diagram depicts the number of gene clusters shared among 43894 and 277. Each circle represents a sample, and the numbers in the circles or the overlapping area denote the number of gene clusters in each strain or shared among the samples.

**Fig. 3.**
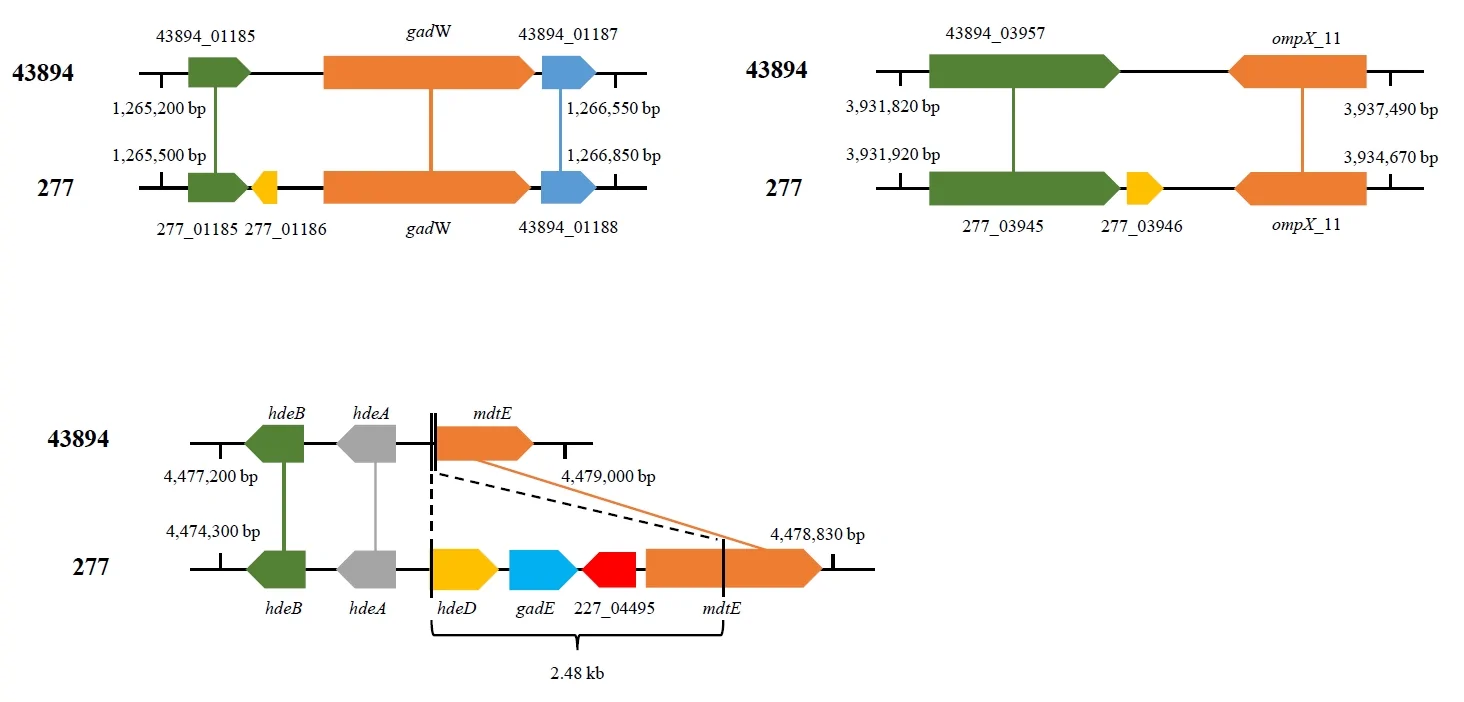
Difference in genetic composition between two EHEC O157:H7 strains 43894 and 277. Gene alignment, curation and comparative sequence analyses of two EHEC strains were performed manually using BLASTn. (A) Identification of hypothetical genes in the strain 277. (B) Deletion of *hdeD*-*gadE*-*mdtE* region in 43894. (C) Genetic variations in the phage-associated genes.

Next, we compared ATCC 43894 and 277 genome sequences to one another to identify genetic differences. As shown in Fig. 2, both strains shared 5323 core genes, whereas 77 genes and 5 genes found only in ATCC 43894 and 277, respectively. Genes found only in ATCC 43894 were closely related to those genes found in pO157, including type II secretion system genes and toxin-antitoxin *ccdA*-*ccdB* (Table S1). Genes found only in 277 were three hypothetical genes, acid-resistance protein *hdeD* and transcription regulator *gadE*. Two hypothetical genes found only in 277 (277_01186 and 277_03946) were located in adjacent to *gadW* and *ompX*, respectively (Fig. 3A). Notably, *hdeD*, *gadE*, a hypothetical gene (277_04495) and a 5′ portion of *mdtE* were deleted in ATCC 43894 genome (Fig. 3B). In addition, several phage related genes present in both strains showed genetic variations (Fig. 3C). Thorough investigations into the gene variations using BLAST revealed that ATCC 43894 genes harbored nucleotide substitutions and frameshift by insertion when compared to other *E. coli* O157:H7 genes, whereas 277 genes were shown to be identical to others (Table 1). These findings suggest that EHEC O157:H7 277 is not isogenic with ATCC 43894, and that malfunction of GDAR system in ATCC 43894 is due to deletion mutation in genes closely related to GDAR regulation, GadE and HdeD.

### Confirmation of *hdeD*-*gadE*-*mdtE* in ATCC 43894 and 277

**Fig. 4.**
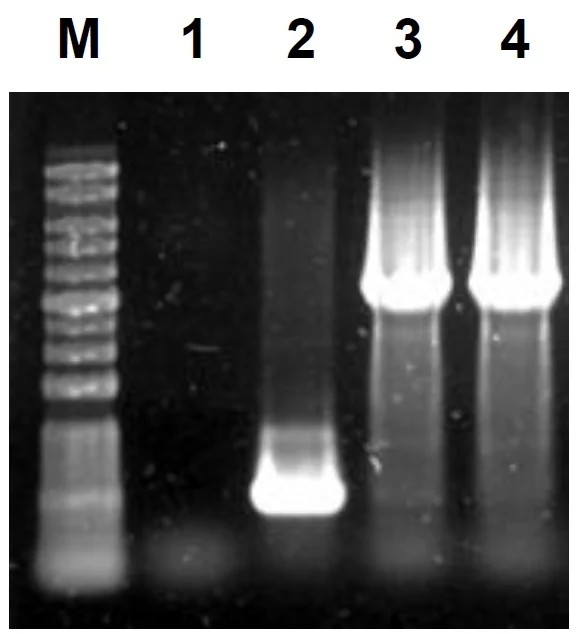
Confirmation of *hdeD*-*gadE*-*mdtE* mutation in EHEC O157:H7 strains 43894 and 277. The *hdeD*-*gadE*-*mdtE* region from the genomic DNA was amplified using two primers targeting sequences adjacent to *hdeD* and *mdtE*. PCR products were visualized on a 2% agarose gel. M, 1.0-kb DNA ladder; 1, negative control; 2, 43894; 3, 277; 4, EDL933.

We further investigated the deletion mutation of *gadE* and *hdeD* found in the whole genome sequencing results. As depicted in Fig. 3B, ATCC 43894 is missing an approximately 2.48-kb region that consists of *hdeD*, *gadE*, a hypothetical gene and a 5′ portion of *mdtE* CDS in its genome as compared to that of in 277. We performed PCR to amplify the missing *hdeD*-*gadE*-*mdtE* region with primers targeting the adjacent sequences to confirm the mutation. As a result, ATCC 43894 formed a 500-bp band whereas other EHEC strains (277 and EDL933) formed approximately a 3.0-kb band (Fig. 4). In addition, amplified bands were ligated to a TA vector, sequenced and identified as the *hdeD*-*gadE*-*mdtE* region (data not shown), indicating that 43894 is missing *gadE* and *hdeD* and that whole genome sequencing performed in this study confirmed genetic variations among two strains.

## Discussion

EDL933 and ATCC 43894 served as reference strains for functional analyses of virulence factors and EHEC pathogenesis. Genetic variations between the reference strains confer various phenotypic traits, adding more complexity in interpretation of given experimental observations. In this study, we discovered an unexpected genetic mutation in a reference strain that had affected the previous observations.

Comparative pangenome analyses revealed that ATCC 43894 is missing two key components of GDAR, *gadE* and *hdeD*. EHEC relies on the GDAR system and a type III secretion apparatus for gut colonization. In *E. coli* strain Sakai, inactivation of *gadE* significantly downregulated many acid fitness island genes, and re-introduction of *gadE* recovered its acid resistance in a simulated gastric environment, clearly demonstrating its significance in bacterial survival (Kailasan Vanaja et al., 2009). Previously, Lim et al. (2007) suggested that pO157 negatively regulates the GDAR system by performing two-dimensional proteomic analyses, but our previous study suggested otherwise that the GDAR genes (*gadA*, *gadB*, *gadC*, *gadW*, and *gadX*) are not regulated by pO157 and the presence of pO157 does not affect GDAR of EHEC 43894 (Wi et al., 2023). Our current findings confirm that pO157 does not harbor regulatory elements that control the GDAR system and suggest that weak acid resistance observed in ATCC 43894 is due to deletion mutation of *gadE* and *hdeD*. This explains the abundance of GadAB in 277 observed in proteome and transcriptome (Lim et al., 2007), as ATCC 43894 had a defect in its expression originally. In addition, this also revealed that pO157 curing in 277 may not have originated from 43894, as *hdeD*-*gadE*-*mdtE* deletion is only present in ATCC 43894. Discrepancy between the two strains should be considered for future interpretation, and this finding also prompts additional investigation of pO157 in *E. coli* O157:H7 for its role in EHEC pathogenesis.

Nucleotide sequence and PCR analysis revealed the deletion of a 2.48-kb fragment which includes *hdeD*, *gadE*, and *mdtE* in ATCC 43894 genome, connecting two truncated genes *hdeD* and *mdtE* to one another (Fig. 3B). This is an unexpected observation as we could not find any potential nucleotide features such as inverted repeats and palindromic sequences adjacent to the deleted region (data not shown). One can postulate that continuous exposure to in vitro environment prompted mutations to gain fitness advantage. Continuous laboratory culture and maintenance may have led to genetic recombination in the genome, as seen in evolution of *A. baumannii* under controlled environment which displayed changes in biofilm formation and virulence (Yun et al., 2024). However, it is not true for ATCC 43894 as genetic recombination has not occurred in this case. In addition to the deletion, hypothetical genes in ATCC 43894 harbored several genetic mutations when compared to their counterparts in 277 (Fig. 3C), including frameshift deletion and nucleotide substitutions (Table 1). These may reflect the genetic variations that are found in other reference strains. Notably, genetic variations in *E. coli* *rpoS* gene are well documented by other research; different strains harbored different types of genetic mutation such as frameshift insertion, deletion, substitution or premature termination of *rpoS* gene. Variations in *rpoS* gene led to differential expressions of biofilm formation and acid resistance patterns in different strains of *E. coli* O157:H7 (Uhlich et al., 2013). We assume that spontaneous mutations may have a role in genetic variations in ATCC 43894.

Our observation also suggests that reference strains may be subjected to genomic diversification during long-term storage. Previously, genomic diversification of laboratory-stored *Salmonella enterica* serovar Typhimurium strain LT2 and LT7 were documented, demonstrating that long-term storage at room temperature in agar stab vials led to phenotypic and genotypic alterations including translocation and point mutations (Edwards et al., 2001; Liu et al., 2003). In addition, Sprouffske and her colleagues observed small changes in *E. coli* genome diversity, with ~1% of rare alleles occurring at low frequencies, but they warned that using glycerol stocks to restart may alter the future evolution of a population if the lost alleles were favorable to the microbe (Sprouffske et al., 2016). We assume that 43894 studied in this work may have favored the loss of *gadE* and *hdeE* under laboratory environment during its evolution. We would also like to provide a caution for others to check uniformity in their archival strains prior to sequencing and experimentation.

Aside from the genetic anomalies, the whole genome sequencing of ATCC 43894 and 277 revealed that the virulence plasmid pO157 was indeed missing from 277, implying that the previous study by Lim et al. (2007) addressed the role of pO157 in EHEC to a certain degree. Although pO157 is apparently not involved in regulation of the GDAR system, pO157-cured 277 showed reduced in vivo colonization and changes in expression of several proteins such as a tryptophanase TnaA. In addition, pO157-cured *E. coli* Sakai strain showed fewer extracellular carbohydrates in biofilm, lower viscosity and less variation in colony morphology (Lim et al., 2010). Other studies also addressed the importance of pO157 in global gene expression, virulence and adaptation of *E. coli* O157:H7 (Tatsuno et al., 2001; Youn et al., 2013). It would be interesting to investigate further into the functional aspects of pO157 in *E. coli* O157:H7 that we may have missed in the previous observation. Taken together, we analyzed the genome sequences of a reference strain EHEC ATCC 43894 and a pO157-cured strain 277 and found that ATCC 43894 harbors genetic mutations affecting glutamate-dependent acid resistance system, implying that the pO157-cured EHEC 277 may not be isogenic to ATCC 43894. This is the first report that such genetic differences between both reference strains of EHEC should be considered in future studies on pathogenic *E. coli*.

## Figures and Tables

**Table 1.** Genetic variations found in ATCC 43894 and 277 phage genes

277 gene	Gene annotation^b^	43894 contig/gene	Mutation	Sequence variation^a^	Note
277_02321	ORM96_01890 (phage portal gene)	43894_02325	Frameshift	c.544_545insA	
		43894 contig 1	Frameshift	g.2314165_2314166insT	
		43894_02324	Frameshift	c.371_372insC	
		43894_02323	Frameshift	c.902_903insA	
277_02325	ORM96_01910 (terminase small subunit)	43894_02331	Frameshift	c.390_391insC	Overlapping with 43894_02330 c.479_480insG
		43894_02330	Frameshift	c.479_480insG	
277_02324	ORM96_01905 (terminase large subunit)	43894_02330	Frameshift	c.56_57insA	
			Frameshift	c.263_264insG	Overlapping with 43894_02331 c.390_391insC
		43894_02329	Frameshift	c.403_404insT	Overlapping with 43894_02328 c.39_40insT
			Substitution	c.488T > C	
			Substitution	c.508A > C	
			Substitution	c.519T > C	
			Substitution	c.525A > G	
			Substitution	c.541T > C	
			Substitution	c.546G > T	
			Substitution	c.558C > T	
			Substitution	c.564G > C	
			Substitution	c.573T > G	
			Substitution	c.576G > T	
			Substitution	c.606G > A	
			Substitution	c.642T > C	
			Substitution	c.666T > G	
			Frameshift	c.946_947insC	
277_02334	ORM96_26320 (DUF1737 domain-containing protein)	43894_02342	Frameshift	c.584_585insG	
		43894_02341	Frameshift	c.926_927insG	
277_02341	ORM96_26280 (RusA family crossover junction endodeoxyribonuclease)	43894_02349	Frameshift	c.187delC	
277_02340	ORM96_26280 (bacteriophage antitermination protein Q)	43894 contig 1	Frameshift	g.2327535_2327536insG	
		43894 contig 1	Frameshift	g.2327438delG	
277_02349	ORM96_26240 (ATP-binding protein)	43894_02359	Frameshift	c.210_211insT	
		43894 contig 1	Frameshift	g.2332332_2332333insG	
277_02350	ORM96_26235 (helix-turn-helix domain-containing protein)	43894_02362	Frameshift	c.274_275insA	Overlapping with 43894_02361 c.6_7insA
		43894_02361	Frameshift	c.6_7insA	Overlapping with 43894_02362 c.274_275insA
			Frameshift	c.285delT	
		43894 contig 1	Frameshift	g.2332991_2332992insC	
		43894_02360	Frameshift	c.35_36insG	

a Genetic mutations and their locations denoted as follows: c, coding sequence; g, genomic DNA; ins, insertion; del, deletion; >, nucleotide substitution.

b Annotated using BLASTn against *E. coli* O157:H7 EDL933 (taxid: 155864) GenBank CP111105.1.
